# Caregiver burden and healthcare providers perspectives in epilepsy: An observational study in China, Taiwan, and Argentina

**DOI:** 10.1016/j.ebr.2024.100736

**Published:** 2024-12-22

**Authors:** Ioannis Karakis, Santiago Flesler, Sanman Ghorpade, Rio Carla Pineda, Kalpesh Joshi, James Cooper, Shilpa Patkar, Andrea Schulz, Savita Bakhshi Anand, Nicola Barnes

**Affiliations:** a Department of Neurology, Emory University School of Medicine, 12 Executive Park Dr NE, Atlanta, GA 30322, USA; b Department of Neurology, University of Crete School of Medicine, Heraklion 71003, Crete, Greece; c Consultant Pediatric Neurologist, Hospital Aleman, CINNES, Av. Pueyrredón 1640, Ciudad de Buenos Aires, Argentina; d Global Medical Affairs, GSK, Dr. Annie Besant Road, Mumbai 400030, Maharashtra, India; e Global Medical Affairs, GSK, 23rd Floor, The Finance Centre, 26th Street, Taguig, 1634 Metro Manila, Philippines; f Global Medical Affairs, GSK, 980 Great West Rd, London TW8 9GS, UK; g Evidera, Inc., 7101 Wisconsin Ave # 1400, Bethesda, MD 20814, USA; h Evidera, Inc., The Ark, 2nd Floor, 201 Talgarth Road, London W6 8BJ, UK

**Keywords:** Epilepsy, Caregiver, Health care providers, Burden

## Abstract

Caregivers are important stakeholders in epilepsy management, and effective communication between healthcare providers (HCPs) and caregivers is needed to optimize patient well-being. The study aimed to identify and evaluate the burden of epilepsy care across the age spectrum from the caregiver’s perspective and understand the perception and management of caregiver burden, including drivers of decision-making in clinical practice, by HCPs. This was a cross-sectional survey of unpaid caregivers of persons with epilepsy (PWE) and HCPs treating PWE. A total of 200 caregivers (China and Taiwan: n = 65/200, 32.5 % each; Argentina: n = 70/200, 35.0 %), and 200 HCPs (China and Taiwan: n = 70/200, 35.0 % each; Argentina: n = 60/200, 30.0 %) participated. Caregivers commonly experienced emotional impacts including anxiety (58.5 %), depression (45.5 %), work/school productivity loss (40.0 %), reduced working hours (40.0 %), or financial stress (24.5 %). HCPs identified several challenges faced by caregivers care, such as fatigue (85.0 %), poor-quality sleep (68.0 %), emotional impacts and stress (61.5 % each), anxiety (57.0 %), and worry about the patient’s future prospects (54.0 %) (e.g., health status (56.5 %), loss of work productivity/career opportunities (50.5 %), worsening of the condition (47.0 %), seizure frequency (46.0 %), and impact on quality of life (46.0 %)). Caregivers for PWE reported emotional, work-related, physical, financial, and social impacts. HCPs recognized similar findings and alignment with the caregiving burden. This study identifies gaps in caregiver support and can be a basis for future initiatives to improve the needs of caregivers and gaps in family-HCP communication so that they can partner with HCPs in the care of PWE.

## Highlights

• Caregivers of PWE reported substantial emotional, physical and social impacts.
• HCPs perspectives aligned with those raised by caregivers on caregiving burden.
• This study identifies gaps in caregiver support and family-HCPs communication.

## 1 Introduction

Epilepsy is one of the most common neurological diseases [1], affecting approximately 50 million individuals worldwide. According to the Global Burden of Epilepsy Report, it is linked to approximately 125,000 deaths per year [2], particularly in developing countries [2]. Worldwide, it is considered one of the major causes of disability and disease-related burden [3]. Specifically, as per the Institute for Health Metrics and Evaluation, it is estimated that 0.52 % of total disability-adjusted life years (DALYs) are attributed to epilepsy [4].

Epilepsy is a disease that impacts more than just the person with the condition, and the caregiver support is paramount in epilepsy management. Epilepsy may contribute largely to the physical and emotional burden not only for patients, but also for caregivers [5], [6]. In particular, it can cause psychosocial distress, disrupt family routines, and reorient the interests and activities of family members. Despite the crucial role of caregivers, much of the literature on epilepsy burden and its impact on quality of life (QoL) has focused on the patients. Information about burden of caring in adult caregivers is scarce.

Likewise, the support caregivers receive from physicians treating persons with epilepsy (PWE) is not well understood. Physicians may find it challenging to provide adequate time for consultation with patients because of a busy clinical practice; therefore, it is not surprising that caregiver concerns often remain unaddressed. Physicians’ knowledge about, attitudes toward, and practices regarding caregiver burden have been insufficiently investigated.

Thus, by drawing from a diverse sample from three countries (China, Taiwan, Argentina) where literature on these topics is scarce, this study aimed to: (1) to identify and evaluate the burden of caring for PWE across the age spectrum from the caregiver’s perspective, and (2) to identify the perception and management of caregiver burden by HCPs as well as the decision-making drivers in clinical practice for managing PWE of all ages.

## 2 Materials and methods

### 2.1 Study design and participants

This was a cross-sectional, non-interventional, descriptive survey study among unpaid caregivers of individuals with epilepsy in China, Taiwan, and Argentina. It also evaluated HCPs with experience treating PWE in the three countries of interest. To be eligible for the study, caregivers were aged at least 18 years, resided in one of the three countries of interest, provided care to a person diagnosed with epilepsy for at least 6 months, did not receive payment for caretaking activities, were able to provide informed consent, were willing to comply with all study procedures, and were able to complete an online survey. The eligibility criteria for HCPs were that they had at least 5 years of experience treating persons diagnosed with epilepsy (including neurologists, epileptologists, and specialty nurses), practiced in one of the three countries of interest, were willing to comply with all study procedures, provided consent, and were able to complete an online survey.

Caregivers and HCPs were recruited by a research vendor specializing in health outcomes research. Participants were asked to complete a self-screener. Once a participant was deemed eligible to participate and had provided electronic informed consent, they were routed to the online survey. Before commencing data collection, ethical approval was obtained in Argentina. China and Taiwan were exempt from ethical review according to local regulations for this form of research. All participants were remunerated for their participation. A summary of the HCP and caregiver enrollment processes can be found in the Supplemental Materials S1. As this was a descriptive study, a target of 200 HCPs and 200 caregivers was considered to be a realistic target for online recruitment in the selected countries and one that would permit the views and perceptions of caregivers and HCPs to be characterized with a satisfactory level of precision. Soft recruitment quotas were put in place to ensure a balanced representation across caregivers and HCPs in each country by age band of the patients they care for or treat and for sub-groups (per country). The precision of estimated response rates was calculated for various sample sizes, but no power analysis was conducted for sample size calculation of this descriptive study. A summary of predicted error margins for different considered sample sizes is provided in the Supplemental Materials S2.

### 2.2 Measures

Two electronic surveys were developed for this study: one for caregivers and another for HCPs. For support in the development of the survey, a steering committee comprising HCPs (n = 3), caregivers (n = 2), and patients (n = 2) was asked to review surveys and supporting background study materials. More specifically, the steering committee members were asked to share their experiences and cultural knowledge, particularly for the survey content and wording. Steering committee member feedback was requested at several stages throughout the study; after the completion of a targeted literature review on epilepsy to identify unaddressed areas of epilepsy caregiving burden, the pilot survey questions were drafted, and the pilot interviews were completed.

The surveys were translated into Spanish for Argentina and Mandarin for China and Taiwan. The research vendor translated, programmed, and hosted all surveys. All study procedures complied with the relevant local regulators, wherever applicable.

Prior to the study launch, a pilot study was completed to test the survey questions drafted for the study. Three caregivers and three HCPs from the three countries of interest were enrolled to complete the survey and provide suggestions for improvement. Following survey completion, caregivers and HCPs in the pilot study participated in one-on-one, 60-minute telephone interviews. Interviews assessed the relevance, comprehensiveness, and understandability of the items and response options. Caregiver interviews were completed in the participant native languages by trained scientific staff. HCP pilot interviews were conducted in English. Most of the questions in the pilot study remained unchanged for the main study. Some items were rephrased or edited to provide missing information or clarity. The eligibility criteria for pilot participants were the same as for the main study. User acceptance testing was completed for both surveys prior to the pilot interviews. All the steps for the pilot and main studies are summarized in the Supplemental Materials S3 and S4, respectively.

The final caregiver survey captured sociodemographic information about the caregiver, general information about the PWE for whom they provide care, information about support used to care for the PWE and impacts on their QoL as a result of caring for the PWE. The final HCP survey collected information on clinical experience; information on the decision-making drivers in clinical practice for managing adults, adolescents, and children with epilepsy; and perceptions about how caregivers of PWE can be better supported to manage their responsibilities. The full primary endpoints addressed in the surveys and the final caregiver and HCP surveys can be found in the Supplemental Materials S5 and S6, respectively.

### 2.3 Data analysis

All survey data was captured electronically by the research vendor. At the conclusion of the data collection, descriptive data analyses were conducted using SAS version 9.0 (Cury, NC). The analyses were only descriptive in nature and no statistical analyses were performed. Continuous variables were summarized using means and standard deviations, and categorical variables were summarized using percentages and frequencies. Subgroup analyses were conducted for caregivers of different pediatric age groups and per country.

## 3 Results

### 3.1 Caregiver results

A total of 200 caregivers (mean age 42 years, 74 % female) completed the survey across China (n = 65/200, 32.5 %), Taiwan (n = 65/200, 32.5 %), and Argentina (n = 70/200, 35.0 %) (Table 1). On average, these participants had been caregivers for 5.8 years and the majority (42.5 %) reported being the patient’s parent.

**Table 1:** Caregiver sociodemographic characteristics.

**Characteristic**	**Total (N = 200)**	**Argentina (n = 70)**	**Taiwan (n = 65)**	**China (n = 65)**
**Gender of caregiver, n (%)**				
Female	148 (74.0 %)	57 (81.4 %)	43 (66.2 %)	48 (73.8 %)
**Caregiver residing with person for whom they cared, n (%)**				
Yes	178 (89.0 %)	49 (70.0 %)	65 (100 %)	64 (98.5 %)
No	22 (11.0 %)	21 (30.0 %)	0 (0.0 %)	1 (1.5 %)
**Age of caregiver**				
Mean (SD)	41.7 (9.2)	40.8 (11.2)	45.4 (7.7)	39.1 (6.9)
Median [range: min–max]	40.0 [23.0–75.0]	38.5 [23.0–75.0]	46.0 [27.0–62.0]	38.0 [25.0–62.0]
**Years as a caregiver**				
Mean (SD)	5.8 (5.2)	8.5 (7.1)	3.6 (2.3)	5.2 (3.6)
Median [range: min–max]	4.6 [0.7–40.9]	6.1 [0.9–40.9]	3.4 [0.7–10.4]	4.4 [1.0–22.8]
**Employment status, n (%)**				
Employed, full-time	97 (48.5 %)	11 (15.7 %)	43 (66.2 %)	43 (66.2 %)
Employed, part-time	44 (22.0 %)	30 (42.9 %)	13 (20.0 %)	1 (1.5 %)
**Highest level of education, n (%)**				
High school	57 (28.5 %)	31 (44.3 %)	13 (20.0 %)	13 (20.0 %)
Attended or finished college/university	129 (64.5 %)	36 (51.4 %)	42 (64.6 %)	51 (78.5 %)
**Relationship with person under care, n (%)**				
The person's parent	85 (42.5 %)	20 (28.6 %)	38 (58.5 %)	27 (41.5 %)
The person's sibling	16 (8.0 %)	11 (15.7 %)	5 (7.7 %)	0 (0.0 %)
The person's child	60 (30.0 %)	6 (8.6 %)	19 (29.2 %)	35 (53.8 %)

Caregivers were asked about the level of assistance the PWE that they care for needed for a range of activities (Table 2). Caregivers most frequently reported that the patient under care needed ‘a little bit of help (supervision only)’ for eating (n = 86/200, 43.0 %), assistance with personal care (n = 71/200, 35.5 %), bowel and bladder management (n = 75/200, 37.5 %), communication (n = 78/200, 39.0 %), and activities at home, school, or work (n = 87/200, 43.5 %). For mobility, the most frequent response given by caregivers in the full sample was that the person does not need help (n = 92/200, 46.0 %). No remarkable differences were found between the countries for these activities. The caregiver level of assistance is also provided by age group in the Supplemental Materials S7. It was reported that higher proportions of children and older adults needed ‘a lot of help’ compared with adolescents and adults for various activities.

**Table 2:** Caregiver level of assistance provided by country.

**Level of Assistance Caregiver Needed**	**Total (N = 200)**	**Argentina (n = 70)**	**Taiwan (n = 65)**	**China (n = 65)**
**Eating, n (%)**				
Does not need help	49 (24.5 %)	23 (32.9 %)	17 (26.2 %)	9 (13.8 %)
Needs a little bit of help − supervision only	86 (43.0 %)	24 (34.3 %)	24 (36.9 %)	38 (58.5 %)
Needs some help	34 (17.0 %)	8 (11.4 %)	14 (21.5 %)	12 (18.5 %)
Needs a lot of help	30 (15.0 %)	14 (20.0 %)	10 (15.4 %)	6 (9.2 %)
Cannot do this at all without help	1 (0.5 %)	1 (1.4 %)	0 (0.0 %)	0 (0.0 %)
**Personal care, n (%)**				
Does not need help	48 (24.0 %)	23 (32.9 %)	16 (24.6 %)	9 (13.8 %)
Needs a little bit of help − supervision only	71 (35.5 %)	19 (27.1 %)	23 (35.4 %)	29 (44.6 %)
Needs some help	47 (23.5 %)	10 (14.3 %)	16 (24.6 %)	21 (32.3 %)
Needs a lot of help	30 (15.0 %)	14 (20.0 %)	10 (15.4 %)	6 (9.2 %)
Cannot do this at all without help	4 (2.0 %)	4 (5.7 %)	0 (0.0 %)	0 (0.0 %)
**Bowel and bladder management, n (%)**				
Does not need help	72 (36.0 %)	31 (44.3 %)	25 (38.5 %)	16 (24.6 %)
Needs a little bit of help − supervision only	75 (37.5 %)	18 (25.7 %)	22 (33.8 %)	35 (53.8 %)
Needs some help	32 (16.0 %)	12 (17.1 %)	8 (12.3 %)	12 (18.5 %)
Needs a lot of help	18 (9.0 %)	6 (8.6 %)	10 (15.4 %)	2 (3.1 %)
Cannot do this at all without help	3 (1.5 %)	3 (4.3 %)	0 (0.0 %)	0 (0.0 %)
**Mobility, n (%)**				
Does not need help	92 (46.0 %)	34 (48.6 %)	37 (56.9 %)	21 (32.3 %)
Needs a little bit of help − supervision only	65 (32.5 %)	17 (24.3 %)	12 (18.5 %)	36 (55.4 %)
Needs some help	30 (15.0 %)	13 (18.6 %)	9 (13.8 %)	8 (12.3 %)
Needs a lot of help	11 (5.5 %)	4 (5.7 %)	7 (10.8 %)	0 (0.0 %)
Cannot do this at all without help	2 (1.0 %)	2 (2.9 %)	0 (0.0 %)	0 (0.0 %)
**Communication, n (%)**				
Does not need help	55 (27.5 %)	29 (41.4 %)	10 (15.4 %)	16 (24.6 %)
Needs a little bit of help − supervision only	78 (39.0 %)	17 (24.3 %)	23 (35.4 %)	38 (58.5 %)
Needs some help	46 (23.0 %)	16 (22.9 %)	21 (32.3 %)	9 (13.8 %)
Needs a lot of help	17 (8.5 %)	5 (7.1 %)	10 (15.4 %)	2 (3.1 %)
Cannot do this at all without help	4 (2.0 %)	3 (4.3 %)	1 (1.5 %)	0 (0.0 %)
**Activities at home, school, or work, n (%)**				
Does not need help	34 (17.0 %)	14 (20.0 %)	7 (10.8 %)	13 (20.0 %)
Needs a little bit of help − supervision only	87 (43.5 %)	28 (40.0 %)	23 (35.4 %)	36 (55.4 %)
Needs some help	56 (28.0 %)	15 (21.4 %)	29 (44.6 %)	12 (18.5 %)
Needs a lot of help	21 (10.5 %)	12 (17.1 %)	5 (7.7 %)	4 (6.2 %)
Cannot do this at all without help	2 (1.0 %)	1 (1.4 %)	1 (1.5 %)	0 (0.0 %)
**Hours per week spent caregiving, n (%)**				
≤8 h	8 (4.0 %)	3 (4.3 %)	1 (1.5 %)	4 (6.2 %)
9–18 h	37 (18.5 %)	12 (17.1 %)	2 (3.1 %)	23 (35.4 %)
19–26 h	33 (16.5 %)	10 (14.3 %)	2 (3.1 %)	21 (32.3 %)
27–36 h	40 (20.0 %)	11 (15.7 %)	20 (30.8 %)	9 (13.8 %)
37–45 h	36 (18.0 %)	7 (10.0 %)	26 (40.0 %)	3 (4.6 %)
≥46 h	46 (23.0 %)	27 (38.6 %)	14 (21.5 %)	5 (7.7 %)
**Time spent by HCP at medical appointments, n (%)**				
<5 min	18 (9.0 %)	2 (2.9 %)	0 (0.0 %)	16 (24.6 %)
5–10 min	19 (9.5 %)	3 (4.3 %)	11 (16.9 %)	5 (7.7 %)
11–15 min	66 (33.0 %)	21 (30.0 %)	26 (40.0 %)	19 (29.2 %)
>15 min	97 (48.5 %)	44 (62.9 %)	28 (43.1 %)	25 (38.5 %)

Caregiving responsibilities were mostly shared with the caregiver’s spouse/partner (n = 132/200, 66.0 %), followed by their parent (n = 40/200, 20.0 %) or sibling (n = 31/200, 15.5 %). Some caregivers reported that they were the sole caregiver for the patient (n = 17/200, 8.5 %). Nearly a quarter of the total sample (n = 46/200, 23.0 %) reported spending ≥ 46 h per week providing care, with a higher proportion of caregivers in Argentina (n = 27/70, 38.6 %) selecting this response compared with caregivers in Taiwan (n = 14/65, 21.5 %) and China (n = 5/65, 7.7 %).

Almost all caregivers reported that they travel to medical appointments of their patients, either always (n = 177/200, 88.5 %) or sometimes (n = 21/200, 10.5 %). Most caregivers also reported staying during appointments (n = 181/200, 90.5 %). Over three-quarters of caregivers reported preparing questions for the doctor (n = 166/200, 83.0 %) and/or completing seizure diaries (n = 152/200, 76.0 %), with two-thirds also preparing medicine/prescriptions/paperwork (n = 133/200, 66.5 %). For those caregivers who accompanied the patient with epilepsy to medical visits, most reported spending more than 10 min with HCPs during appointments (Table 2). The burden of caregiving and medical appointments based on PWE age group is presented in the Supplemental Materials S8.

The majority of caregivers confirmed that they were involved in treatment decisions (n = 171/200, 85.5 %). Less than half of the caregivers (n = 86/200, 43.0 %) reported that they could discuss any topic with HCPs. The most commonly reported uncomfortable topics for the caregiver to bring up (as it relates to the patient) were sleep (n = 51/200, 25.5 %), work (n = 44/200, 22.0 %), and sexual activity (n = 40/200, 20.0 %). The most frequently reported uncomfortable topics for the patient to discuss were behavioral problems (n = 48/200, 24.0 %), cognitive problems (n = 45/200, 22.5 %), emotional/psychological issues (n = 42/200, 21.0 %), and sexual activity (n = 32/200, 16.0 %).

**Fig. 1:**
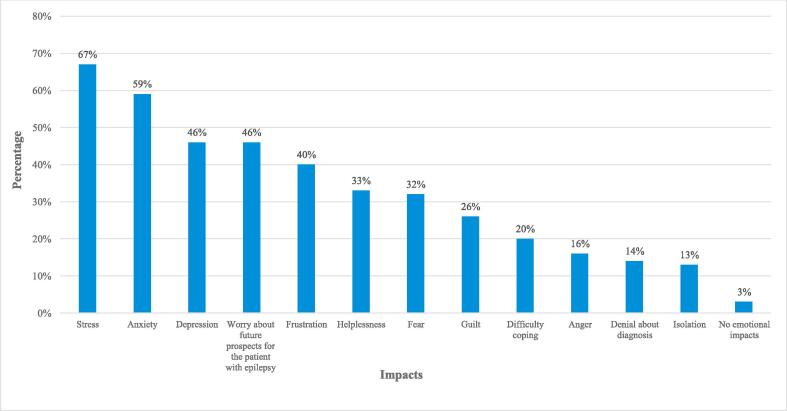
Emotional impacts reported by caregivers.

Caregivers reported considerable psychosocial, emotional, mental, physical, financial, social and work-related impacts. These impacts are summarized in the Supplemental Materials S9 and S10, and a graphical presentation is provided in Fig. 1. A significant proportion of caregivers reported stress (n = 133/200, 66.5 %), anxiety (n = 117/200, 58.5 %) and depression (n = 91/200, 45.5 %) because of caregiving. Other frequently reported impacts included not being as productive at work/school (n = 80/200, 40.0 %), having to adjust their working hours (n = 80/200, 40.0 %), or experiencing financial stress (n = 49/200, 24.5 %; Supplemental Materials S10). Of these, productivity at work/school was consistently among the top three impacts experienced by caregivers in all three countries (see Supplemental Materials S11 for factors interfering with work/study). Adapting work hours was infrequently selected by caregivers in China (n = 8/65, 12.3 %) compared with those in Taiwan (n = 39/65, 60.0 %) and Argentina (n = 33/70, 47.1 %), and financial stress was selected more often by caregivers in Argentina (n = 23/70, 32.9 %) compared with those in Taiwan and China (n = 13/65, 20.0 % each). Additionally, most caregivers had experienced some degree of fatigue over the previous week, with the majority experiencing at least 3 days of feeling fatigued (n = 82/200, 41.0% fatigued for 3 to 5 days; n = 43/200, 21.5% fatigued all the time; Supplemental Materials S9 and S12). Sleep interruptions over the past week were also reported by the majority of caregivers (Supplemental Materials S9 and S13). Furthermore, caregivers reported difficulties in enrolling PWE in activities, mainly due to there being no special instructors dedicated to PWE with special needs and due to educational centers for PWE with special needs being too expensive (Supplemental Materials S9 and S14).

The most commonly reported coping strategies caregivers used to mitigate such impacts included learning about the condition so that they know what to do (n = 141/200, 70.5 %), doing their best in carrying out their caregiving responsibilities and leaving the rest to the doctors (n = 130/200, 65.0 %), and having their family to support them when they need it (n = 116/200, 58.0 %; Supplemental Materials S9 and S15).

In general, caregivers reported that the most relevant information for them was about epilepsy (n = 184/200, 92.0 %), treatments (n = 156/200, 78.0 %), and psychological support services for themselves (n = 109/200, 54.5 %) (see Supplemental Materials S16). Slightly more than half of the caregivers were satisfied with the information they received. The most common emotional support networks included family members or friends (n = 138/200, 69.0 %), medical professionals (n = 73/200, 36.5 %), and mental health support groups (n = 35/200, 17.5 %; Supplemental Materials S16). Support from family members and medical professionals was consistently within the top three responses in all three countries. Notably, caregivers in Taiwan almost exclusively selected religious groups for emotional support (n = 26/65, 40.0 %; Supplemental Materials S16).

The top three concerns expressed by the caregiver about outlook of PWE were future health (n = 113/200, 56.0 %), seizures becoming worse in the future (n = 94/200, 47.0 %), seizures occurring more often in the future (n = 92/200, 46.0 %), and the QoL of the PWE (n = 92/200, 46.0 %). Almost all caregivers indicated assisting the PWE in taking their medication (n = 195/200, 97.5 %). The person under care took medication as prescribed all of the time (n = 169/200, 84.5 %) or most of the time (n = 21/200, 10.5 %).

### 3.2 HCP results

A total of 200 HCPs completed the survey across China (n = 70/200, 35.0 %), Taiwan (n = 70/200, 35.0 %), and Argentina (n = 60/200, 30.0 %) (Table 3). HCPs most frequently reported 11 to 20 years (n = 81/200, 40.5 %) of experience treating PWE, and nearly three-quarters of HCPs indicated their specialty as general neurologists (n = 147/200, 73.5 %). Table 3 summarizes further HCPs information including practice type and location, populations treated, and services rendered. HCPs spent the longest routine appointment time with older adults aged ≥ 65 years, and HCPs in Taiwan spent the least amount of time with all patient categories compared with HCPs in Argentina and China (Table 3).

**Table 3:** HCP characteristics.

**Characteristic**	**Total (N = 200)**	**Argentina (n = 60)**	**Taiwan (n = 70)**	**China (n = 70)**
**Years of experience, n (%)**				
5–10	80 (40.0 %)	32 (53.3 %)	16 (22.9 %)	32 (45.7 %)
11–20	81 (40.5 %)	16 (26.7 %)	34 (48.6 %)	31 (44.3 %)
≥21	39 (19.5 %)	12 (20.0 %)	20 (28.6 %)	7 (10.0 %)
**Specialty^a^, n (%)**				
Neurologist	147 (73.5 %)	45 (75.0 %)	70 (100.0 %)	32 (45.7 %)
Pediatric	26 (17.7 %)	10 (22.2 %)	13 (18.6 %)	3 (9.4 %)
Adolescent	49 (33.3 %)	18 (40.0 %)	20 (28.6 %)	11 (34.4 %)
Adult	127 (86.4 %)	38 (84.4 %)	58 (82.9 %)	31 (96.9 %)
Epileptologist	63 (31.5 %)	25 (41.7 %)	7 (10.0 %)	31 (44.3 %)
Epilepsy specialist nurse	26 (13.0 %)	8 (13.3 %)	0 (0.0 %)	18 (25.7 %)
**Practice type^a^, n (%)**				
Government/public hospital/clinic	114 (57.0 %)	32 (53.3 %)	14 (20.0 %)	68 (97.1 %)
Private hospital	80 (40.0 %)	29 (48.3 %)	51 (72.9 %)	0 (0.0 %)
Private practice/clinic	41 (20.5 %)	41 (68.3 %)	0 (0.0 %)	0 (0.0 %)
Research/university hospital	27 (13.5 %)	6 (10.0 %)	13 (18.6 %)	8 (11.4 %)
**Outpatient proportion, n (median)**	200 (70.0)	60 (80.0)	70 (80.0)	70 (50.0)
**Inpatient proportion, n (median)**	200 (30.0)	60 (20.0)	70 (20.0)	70 (50.0)
**Main place of work, n (%)**				
City center	166 (83.0 %)	52 (86.7 %)	53 (75.7 %)	61 (87.1 %)
City suburbs	21 (10.5 %)	3 (5.0 %)	12 (17.1 %)	6 (8.6 %)
**Online and/or telephone appointments**				
**Yes, I use this service, n (%)**	140 (70.0 %)	41 (68.3 %)	62 (88.6 %)	37 (52.9 %)
Percentage of appointments online or by telephone, mean (SD)	16.3 (19.0)	21.2 (17.7)	12.2 (21.6)	17.8 (14.4)
Percentage of urgent inquiries, mean (SD)	13.6 (17.9)	22.4 (21.7)	3.5 (6.4)	20.9 (18.0)
Percentage of time caregivers taking part in conversations during online or telephone, mean (SD)	45.1 (30.2)	53.5 (28.1)	38.0 (33.5)	47.7 (24.0)
**Yes, but I do not use this service, n (%)**	39 (19.5 %)	12 (20.0 %)	7 (10.0 %)	20 (28.6 %)
Persons with epilepsy do not like virtual appointments, n (%)	6 (15.4 %)	1 (8.3 %)	1 (14.3 %)	4 (20.0 %)
I do not like virtual/telephone appointments, n (%)	13 (33.3 %)	4 (33.3 %)	4 (57.1 %)	5 (25.0 %)
I prefer to see patients face to face and to conduct examinations if needed, n (%)	32 (82.1 %)	11 (91.7 %)	4 (57.1 %)	17 (85.0 %)
**Patients seen per week, n (%)**				
<20	83 (41.5 %)	23 (38.3 %)	34 (48.6 %)	26 (37.1 %)
21–40	89 (44.5 %)	31 (51.7 %)	28 (40.0 %)	30 (42.9 %)
41–60	20 (10.0 %)	5 (8.3 %)	5 (7.1 %)	10 (14.3 %)
**Patient age groups, n (%)**				
Pediatrics (birth to 11 years)	60 (30.0 %)	18 (30.0 %)	14 (20.0 %)	28 (40.0 %)
Adolescents (12–17 years)	131 (65.5 %)	44 (73.3 %)	36 (51.4 %)	51 (72.9 %)
Adults (18–64 years)	181 (90.5 %)	55 (91.7 %)	63 (90.0 %)	63 (90.0 %)
Older Adults (≥65 years)	166 (83.0 %)	50 (83.3 %)	56 (80.0 %)	60 (85.7 %)
**Time spent (in minutes) with patients during routine appointments, mean (SD)**				
Adults (18–64 years)	16.9 (15.7)	26.1 (20.5)	8.8 (9.9)	17.1 (10.6)
Adults (≥65 years)	19.5 (19.2)	29.1 (23.9)	11.0 (16.3)	19.7 (12.3)
Adolescents (12–17 years)	16.9 (15.8)	25.2 (19.9)	7.7 (10.0)	19.0 (11.6)
Pediatrics (birth to 11 years)	12.9 (18.0)	14.4 (23.9)	5.3 (9.8)	19.2 (15.6)

**^a^**Responses are not mutually exclusive.

**Fig. 2:**
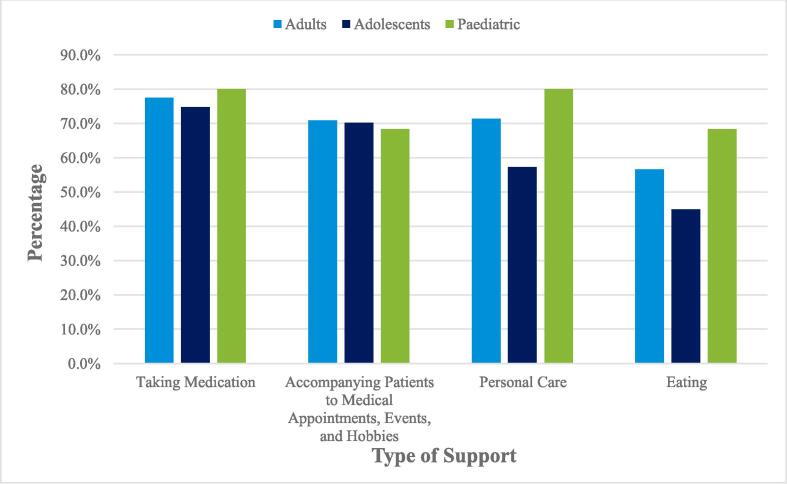
Frequently reported types of significant caregiver support required by patients as described by healthcare professionals.

The most frequently reported types of support PWE required as per their HCPs were taking antiseizure medication (ASMs); accompanying patients to medical appointments, events, and/or hobbies; personal care, and eating (Fig. 2). Pediatric patients consistently required the greatest amount of support compared with adult and adolescent patients (≥5% differences) for eating, personal care, bowel and bladder management, communicating, activities at home, and taking medication (see Supplemental Materials S17). However, support required for taking ASMs and accompanying patients to medical appointments was similar for all age groups of patients according to the overall HCP population (Fig. 2).

HCPs reported that caregivers attend appointments, either always or sometimes for adults (n = 98/200, 49.0 % and n = 96/200, 48.0 %, respectively), adolescents (n = 145/200, 72.5 % and n = 45/200, 22.5 %, respectively), and pediatric patients (n = 161/200, 80.5 % and n = 12/200, 6.0 %, respectively). The majority of HCPs also confirmed that caregivers typically led conversations during appointments for the epilepsy patients and were either always or frequently involved in treatment decisions, irrespective of the patient’s age.

Overall, most HCPs reported that caregivers discussed issues with them related to caregiving for PWE always or sometimes (n = 186/200, 93.0 %), citing a wide range of impacts experienced by caregivers (Table 4). The most common caregiving impacts reported to them included fatigue (n = 170/200, 85.0 %), poor-quality sleep (n = 136/200, 68.0 %), and generalized emotional impacts and stress (n = 123/200, 61.5 % each). HCPs felt uncomfortable discussing certain topics with caregivers including sexual activity (n = 94/200, 47.0 %), illicit drug use (n = 52/200, 26.0 %), sudden unexplained death of epilepsy patients (n = 51/200, 27.5 %) and emotional/psychological issues (e.g., anxiety or depression (n = 42/200, 21.0 %)).

**Table 4:** HCP-reported caregiver impacts by country.

**Characteristic**	**Total (N = 200)**	**Argentina (n = 60)**	**Taiwan (n = 70)**	**China (n = 70)**
**Do caregivers discuss their own problems, n (%)**				
Yes	127 (63.5 %)	27 (45.0 %)	45 (64.3 %)	55 (78.6 %)
No	14 (7.0 %)	5 (8.3 %)	5 (7.1 %)	4 (5.7 %)
Sometimes	59 (29.5 %)	28 (46.7 %)	20 (28.6 %)	11 (15.7 %)
**Do caregivers discuss experiencing the following^a^, n (%)**				
Caregiver’s fatigue	170 (85.0 %)	49 (81.7 %)	63 (90.0 %)	58 (82.9 %)
Caregiver’s poor sleep quality	136 (68.0 %)	39 (65.0 %)	50 (71.4 %)	47 (67.1 %)
Caregiver’s job loss/productivity loss/ career opportunity loss/ limitations on number of hours worked/ job security	101 (50.5 %)	26 (43.3 %)	43 (61.4 %)	32 (45.7 %)
Emotional impacts on caregivers	123 (61.5 %)	34 (56.7 %)	56 (80.0 %)	33 (47.1 %)
Anxiety	114 (57.0 %)	29 (48.3 %)	45 (64.3 %)	40 (57.1 %)
Depression	82 (41.0 %)	26 (43.3 %)	35 (50.0 %)	21 (30.0 %)
Stress	123 (61.5 %)	41 (68.3 %)	55 (78.6 %)	27 (38.6 %)
Difficulty coping	66 (33.0 %)	25 (41.7 %)	25 (35.7 %)	16 (22.9 %)
Guilt	27 (13.5 %)	14 (23.3 %)	7 (10.0 %)	6 (8.6 %)
Helplessness	62 (31.0 %)	16 (26.7 %)	27 (38.6 %)	19 (27.1 %)
Isolation	35 (17.5 %)	9 (15.0 %)	14 (20.0 %)	12 (17.1 %)
Anger	24 (12.0 %)	5 (8.3 %)	11 (15.7 %)	8 (11.4 %)
Fear	32 (16.0 %)	15 (25.0 %)	10 (14.3 %)	7 (10.0 %)
Worry about future prospects for the person with epilepsy	108 (54.0 %)	32 (53.3 %)	44 (62.9 %)	32 (45.7 %)
Unknown	0 (0.0 %)	0 (0.0 %)	0 (0.0 %)	0 (0.0 %)
Family relations	73 (36.5 %)	22 (36.7 %)	30 (42.9 %)	21 (30.0 %)
Caregiver’s social life	79 (39.5 %)	27 (45.0 %)	29 (41.4 %)	23 (32.9 %)
Finances	86 (43.0 %)	25 (41.7 %)	42 (60.0 %)	19 (27.1 %)
None of the above	1 (0.5 %)	1 (1.7 %)	0 (0.0 %)	0 (0.0 %)
Stigma	19 (9.5 %)	4 (6.7 %)	9 (12.9 %)	6 (8.6 %)
**Type of support sought by caregivers^a^, n (%)**				
Emotional or moral support	155 (77.5 %)	51 (85.0 %)	54 (77.1 %)	50 (71.4 %)
Financial support	98 (49.0 %)	25 (41.7 %)	32 (45.7 %)	41 (58.6 %)
Support with decisions about treatments	111 (55.5 %)	19 (31.7 %)	41 (58.6 %)	51 (72.9 %)
Practical support	76 (38.0 %)	36 (60.0 %)	10 (14.3 %)	30 (42.9 %)
Other	0 (0.0 %)	0 (0.0 %)	0 (0.0 %)	0 (0.0 %)
I don’t know	9 (4.5 %)	1 (1.7 %)	7 (10.0 %)	1 (1.4 %)

**^a^**Responses are not mutually exclusive.

HCPs reported that the most common caregiver concerns for patients included uncontrollable seizures (n = 164/200, 82.0 %), intellectual/behavioral impairments (n = 100/200, 50.0 %), uncertainty about future (n = 97/200, 48.5 %), issues with job/schooling performance (n = 92/200, 46.0 %), dependency on ASMs (n = 83/200, 41.5 %), social acceptance (n = 74/200, 37.0 %), and dependency on caregivers (n = 70/200, 35.0 %).

Similar to caregivers, HCPs were asked about the type of support caregivers sought for caring for a PWE (Table 4). More than three-quarters of the HCPs sample (n = 155/200, 77.5 %) reported that caregivers looked for emotional or moral support.

HCPs indicated they most commonly advise caregivers to ask questions (e.g., during medical appointments; n = 168) and to seek written information (e.g., printed leaflets; n = 128), information from a named person from the epilepsy team at the hospital (e.g., a nurse; n = 55), information from other people who care for someone with epilepsy (n = 54), or information from online groups (e.g., Facebook; n = 50) as sources of information and support (see Supplemental Materials S18).

## 4 Discussion

The findings in this study illustrate a detailed landscape of caregiver concerns relating to caring for PWE in Argentina, China, and Taiwan. The humanistic and economic burden of caregiving in epilepsy has previously been reported in both qualitative and quantitative studies in other countries [7], [8], [9] and for specific types of the disease. In our study, we surveyed both HCPs and caregivers to document experience and needs from perspectives that are informed by considerably different roles and levels of knowledge.

Overall, caregivers commonly experienced emotional, work-related, physical, financial and social impacts, which were also recognized by HCPs. Most caregivers reported that they travelled to and stayed for medical appointments of PWE, that they were involved in treatment decisions and that they commonly assisted PWE in taking their medication. The main concerns of caregivers were related to future health and QoL of PWE. Similar findings were reported by HCPs: they reported that support for taking ASMs and accompanying patients to medical appointments was similar for all age groups of patients, and confirmed that caregivers typically led conversations during appointments and were involved in treatment decisions. Most HCPs reported that caregivers discussed issues with them related to caregiving for PWE and that they looked for emotional or moral support.

There were no marked differences in the level of assistance provided by caregivers between participating countries; however, caregivers in Argentina reported more time spent providing care and higher financial stress compared with caregivers in China or Taiwan. Caregivers in Argentina and Taiwan reported having to change their work hours more frequently than those in China. In Taiwan, HCPs spent less time with patients compared with HCPs in the other countries, and caregivers frequently sought emotional support from religious groups. Better understanding of the challenges faced by caregivers in these countries, as well as the help they receive, is needed and is a key step towards providing relevant support for these populations.

As shown in our study, caregivers spent considerable amount of time caring for PWE: nearly a quarter of caregivers reported spending ≥ 46 h a week providing caregiving activities. A recent study in Malaysia [7] cited a mean weekly caregiving time of over 77 h per week, whereas a study in six European countries [10] found that the most common time spent per week was 25 to 34 h. Past research has also shown that an increase in time spent with a patient is associated with a higher burden of caring for patients experienced by the caregiver [5].

Due to their poor recollection of their seizures and potential cognitive difficulties at baseline, PWE rely on caregivers to help them with ASMs compliance, transportation to their medical appointments, engagement with HCPs, personal care, eating support, communication support, among others. The toll on the caregivers is wide-ranging, from frequent emotional impacts, such as stress, anxiety, and depression, to fatigue, sleep difficulties, lower productivity, and financial difficulties. Of note, most caregivers in our study were females who had studied at college or university; however, less than half of caregivers had a full-time job. This was particularly noticeable in Argentina and was similar to another study in Malaysia [7], highlighting how caregiving may markedly impact people’s income and earning potential, especially in women. Overall, these findings are consistent with past research [9], [11] that indicates that caregiving duties may pose a significant demand on caregivers’ time and can impact different facets of their life including their emotional and physical health as well as social, work-related, and financial aspects [12].

Our findings showed that both caregivers and HCPs (comprising neurologists, epileptologists, and epilepsy specialist nurses) were aware of the level of burden that is placed upon a caregiver caring for an adult, adolescent, or child with epilepsy, with little discrepancies between the sub-groups (i.e., caregivers and HCPs and the three countries). HCPs reported that the majority of caregivers discuss the problems that they experience due to caring for PWE. Despite this awareness, HCPs may be unable to help caregivers alleviate some of this burden because of limited appointment times, high volumes of patients, and a lack of other resources in their practices. As noted by both caregivers and HCPs in this study, appointments are often relatively short in duration and are mainly focused on the patient’s requirements. Therefore, there may be limited time for discussion about a caregiver’s physical and mental health. Moreover, caregivers and patients may not be willing to discuss certain uncomfortable topics further limiting the assistance they can receive from HCPs. It is also noteworthy that despite huge mental and emotional impacts including stress, anxiety and depression experienced by the caregivers, HCPs may not always be comfortable discussing these topics. These factors highlight the need for integrating multidisciplinary approach while treating epilepsy patients [8].

There is limited written information that can be provided to caregivers about impacts experienced as a result of caring for PWE. In such cases, it may be appropriate for HCPs to provide other resources that may be beneficial for them. For example, the three most relevant types of information for caregivers in the current study were information about epilepsy, information about treatments, and information about psychological support services for themselves. Support from family members and medical professionals was consistently within the top three responses in all three countries, which illustrates the need for this type of information for caregivers. On the other hand, HCPs reported that the sources of information they recommended included seeking oral information (e.g., during medical visits/appointments), written information (e.g., printed leaflets), a named person from the epilepsy team at the hospital (e.g., a nurse), other people who care for someone with epilepsy, or online groups (e.g., Facebook). Indeed, self-management programs generally have been successful in preparing people to manage medical conditions with recurring events [13]. Personalized self-management education programs that allow design input from PWE and their caregivers would allow for the most relevant types of information to be housed in one place (i.e., with the consulting physician) and referred to when needed.

The findings of this study have implications for future clinical practice. Although HCPs do not focus on caregivers as part of their patient-management plan, they are well-placed to understand the different aspects of caregiver burden, to raise awareness, and to direct the caregiver to resources that may help to alleviate the burden of their caretaking responsibilities, shifting the focus of care towards the family as opposed to the person in isolation. Ideally, HCPs could incorporate caregivers into the assessment and treatment care structure and plan; multi-disciplinary clinics where a team of HCPs including social workers and psychologists may help to support caregiver concerns and regularly assess their QoL, with the aim of improving the patient’s QoL in turn. As proposed by past research, this may include providing caregiver resources such as counseling, education, individualized and/or group multidisciplinary interventions, and different types of support to alleviate the burden experienced by caregivers [5]. For example, epilepsy specialist nurses can provide valuable support in educating patients as well as caregivers and much needed physical, emotional and social support, which may in turn help to alleviate some of the burden experienced. Other strategies that have been successful among caregivers of PWE include information linker services where caregivers can submit questions and receive personalized reports [14], and simulation education training to improve self-efficacy of caregivers [15]. With the latest developments in technology, telehealth also plays an important role in supporting caregivers and alleviating some of the burden of caring for children with epilepsy [16], [17]. Other approaches such as caregiver intensive education programs [18], seizure detection devices [19], and stress management courses [20] have also been shown to improve the QoL of both PWE and their caregivers.

Overall, results from our study indicate that caregivers are well-placed to provide valuable input into the treatment plan of PWE and the burden that this places on overall family wellbeing. The perspectives, feelings and needs of caregivers identified in this study should be incorporated into agenda and expert opinion panel reviews of epilepsy advocacy groups to help provide much needed visibility on caregiver burden and potential solutions to address this unmet need. National clinical guidelines and policy recommendations should emphasize caregiver QoL as one of the core quality measures for ensuring holistic outcomes in epilepsy management. The focus of investigation for future research should expand to incorporate family wellbeing. To alleviate some of the burden on PWE and caregivers, policymakers should increase resource allocation and research funding for initiatives aimed at addressing caregiver needs, as well as promote welfare and information programs. Increasing availability of and access to healthcare professionals that can support caregivers, and improvements in information resources for caregivers would go a long way towards improving caregiver wellbeing.

### 4.1 Strengths and limitations

The main strength of this study is the inclusion of caregivers and HCP perspectives from two continents and three culturally distinct countries with a relatively large and equally distributed sample size. Given the lack of information found in the literature on caregiver burden and HCP perspectives, the current study has provided valuable insights into the factors responsible for the burden of caregivers who support PWE across the age spectrum. We acknowledge some limitations typically associated with descriptive surveys. One of the main limitations was that no statistical analyses were performed, and therefore only descriptive comparisons can be made from our data. Although precision estimates were used to calculate the sample size, no power analysis was conducted, which may limit the generalizability due to potential selection and response biases. Like in every survey study, there exists a possibility of selection bias, whereas the experiences and preferences of participants may have been systematically different from the general population of caregivers and HCPs in epilepsy. Additionally, despite all good intents to capture a wide range of perspectives of caregivers and HCPs, there is always a potential for response bias as all the responses were self-reported by the caregivers without formal interviews/investigation in clinic settings. There was no formal quantitative assessment of parameters like anxiety and depression. Furthermore, confirmation of epilepsy diagnosis was not requested from participants; caregivers self-identified as caregivers of PWE to be eligible to participate in the study. A control group (i.e., HCPs and caregivers caring for persons with other chronic neurological disorders) that would allow for comparison of burden between different chronic paroxysmal neurological disorders was not included in our study and can be a subject of future research. Factoring in the advantages of online questionnaires, both the HCP and caregiver surveys were available only electronically. Thus, only those with access to technology (i.e., computer or tablet with internet) were able to participate in the study. Lastly, our assessment was conducted cross-sectionally; hence further evaluations are required to provide a longitudinal insight of our findings and the effect of potential interventions to address them.

Despite the limitations of the survey methodology, additional steps were taken to ensure that the information collected was comprehensive. The final survey was designed after expert panel consensus, and a pilot survey was also conducted prior to the main survey, which allowed to update the survey based on the initial feedback received.

## 5 Conclusions

Findings from caregiver and HCP surveys collected in China, Taiwan, and Argentina indicate that epilepsy caregivers experience a variety of impacts because of their caring activities. The most frequently experienced impacts reported by caregivers were emotional, physical, financial, work, and school-related. HCP responses aligned with the caregivers on their perception of caregiving burden. To help caregivers in their role and to minimize their burden, identifying resources such as a designated liaison from the epilepsy team, provision of written information and engagement in support groups are proposed. This study identifies gaps for caregiver support and family-HCPs collaboration that will allow strategies to be tailored to meet country and population-specific needs.

## 6 Availability of data and materials

The data supporting the current study’s findings are available at reasonable request from the corresponding author. The data are not publicly available because of privacy or ethical restrictions.

## CRediT authorship contribution statement

**Ioannis Karakis:** Writing – review & editing, Study design. **Santiago Flesler:** Writing – review & editing. **Sanman Ghorpade:** Writing – review & editing, Methodology, Conceptualization, Visualization. **Rio Carla Pineda:** Writing – review & editing, Methodology, Conceptualization, Visualization. **Kalpesh Joshi:** Writing – review & editing, Methodology, Conceptualization, Visualization. **James Cooper:** Writing – review & editing, Methodology, Conceptualization, Visualization. **Shilpa Patkar:** Writing – review & editing, Methodology, Conceptualization, Visualization, Formal analysis. **Andrea Schulz:** Project administration, Formal analysis, Visualization, Writing – review & editing, Conceptualization, Methodology. **Savita Bakhshi Anand:** Writing – review & editing, Methodology, Formal analysis, Conceptualization, Visualization, Project administration. **Nicola Barnes:** Writing – review & editing, Methodology, Formal analysis, Conceptualization, Visualization, Project administration.

## Ethics approval

The authors confirm that we have read the journal’s position on issues involved in ethical publication and affirm that the current study is consistent with those guidelines. All participants provided electronic informed consent before participating in any study activities. Ethical approval was obtained in Argentina (approval date: December 27, 2022; reference number: 8548). China and Taiwan were exempt from ethical review according to local regulations.

## Funding

This study (218230) was funded by GSK.

## Declaration of competing interest

The authors declare the following financial interests/personal relationships which may be considered as potential competing interests: Ioannis Karakis reports a relationship with GSK that includes: consulting or advisory. Santiago Flesler reports a relationship with GSK that includes: consulting or advisory. Sanman Ghorpade reports a relationship with GSK that includes: consulting or advisory, employment, and equity or stocks. Rio Carla Pineda reports a relationship with GSK that includes: consulting or advisory, employment, and equity or stocks. Kalpesh Joshi reports a relationship with GSK that includes: consulting or advisory, employment, and equity or stocks. James Cooper reports a relationship with GSK that includes: consulting or advisory, employment, and equity or stocks. Shilpa Patkar reports a relationship with GSK that includes: consulting or advisory, employment, and equity or stocks. Andrea Schulz reports a relationship with GSK that includes: consulting or advisory and funding grants. Savita Bakhshi Anand reports a relationship with GSK that includes: consulting or advisory and funding grants. Nicole Barnes reports a relationship with GSK that includes: consulting or advisory and funding grants. This study (218230) was funded by GSK. Editorial assistance was provided by Evidera Editorial & Design Services. Data collection tools and study design support was provided by Asha Hareendran and Nashmel Sargalo. If there are other authors, they declare that they have no known competing financial interests or personal relationships that could have appeared to influence the work reported in this paper.
