# Supplementary material for: Caregiver burden and healthcare providers perspectives in epilepsy: An observational study in China, Taiwan, and Argentina
Source: Epilepsy Behav Rep. 2024 Dec 22;30:100736. doi: 10.1016/j.ebr.2024.100736 (PMC11928858; doi:10.1016/j.ebr.2024.100736)
Supplement: Supplementary Data 1 [file mmc1.docx]

# SUPPLEMENTAL MATERIALS

S1. Caregiver and HCP Survey Enrolment Summaries


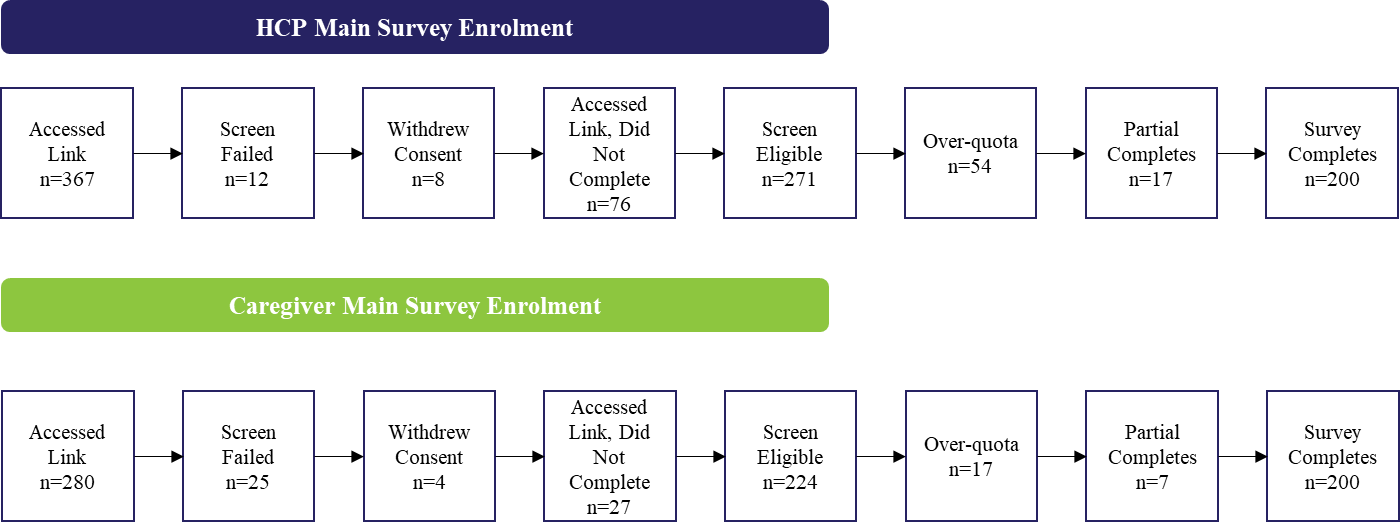


Abbreviations: HCP = healthcare professional

S2. Precision of estimated response rates

A sample of 200 caregivers and 200 healthcare professionals (HCPs) was planned to be sought as a realistic target for on-line recruitment in the selected countries and to permit the views and perceptions of caregivers and HCPs to be satisfactorily characterised with the newly developed study surveys.

The precision of estimated response rates for various sample sizes are shown in Table S2, according to the proportion of participants responding in a particular way to the survey questions. The table summarises the margin of error at the 95% confidence level provided by varying sample sizes and estimates of percentage of participant response. For example, if the estimate of the percentage of HCPs who frequently discuss caregiver resilience during patient consultations was 60%, then a sample of 200 HCPs would provide a margin of error of ± 6.9 percentage points of this estimate with a 95% confidence interval.

| **Sample size** | **Proportion of Participants Responding to Survey Questions with the Same Response** | | | | | | |
| --- | --- | --- | --- | --- | --- | --- | --- |
|  | **50** | **60** | **70** | **75** | **80** | **85** | **90** |
|  | **Precision/ Margin of Error (± %) with 95% confidence interval** | | | | | | |
| 50 | 13.9 | 13.6 | 12.7 | 12 | 11.1 | 9.9 | 8.3 |
| 100 | 9.8 | 9.6 | 9 | 8.5 | 7.8 | 7 | 5.9 |
| 150 | 8 | 7.8 | 7.3 | 6.9 | 6.4 | 5.7 | 4.8 |
| 200 | 6.9 | 6.8 | 6.4 | 6 | 5.5 | 4.9 | 4.2 |
| 250 | 6.2 | 6.1 | 5.7 | 5.4 | 5 | 4.4 | 3.7 |
| 300 | 5.7 | 5.5 | 5.2 | 4.9 | 4.5 | 4 | 3.4 |

**S3. Summary of Pilot Study**


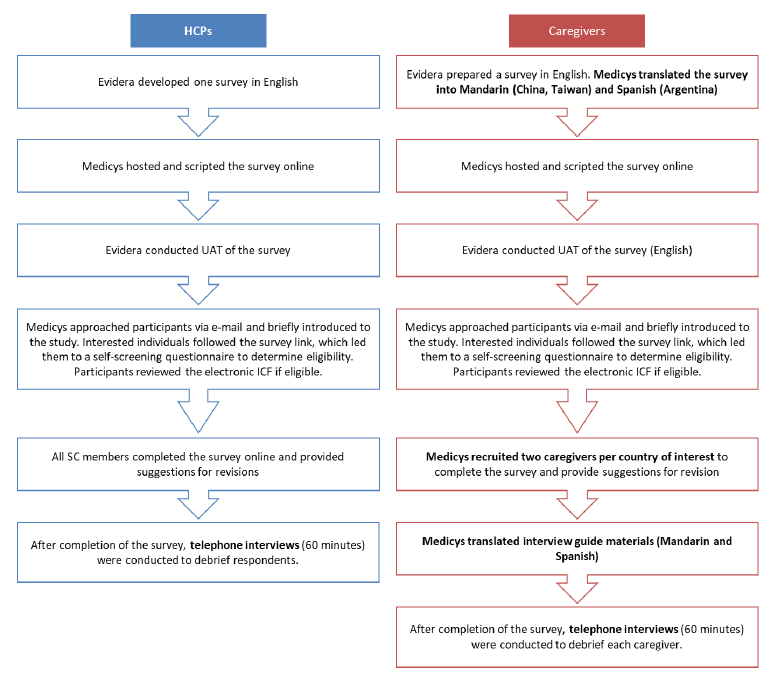


Abbreviations: HCP = healthcare professional; ICF = informed consent form; UAT = user acceptance testing

**S4. Summary of Main Study**


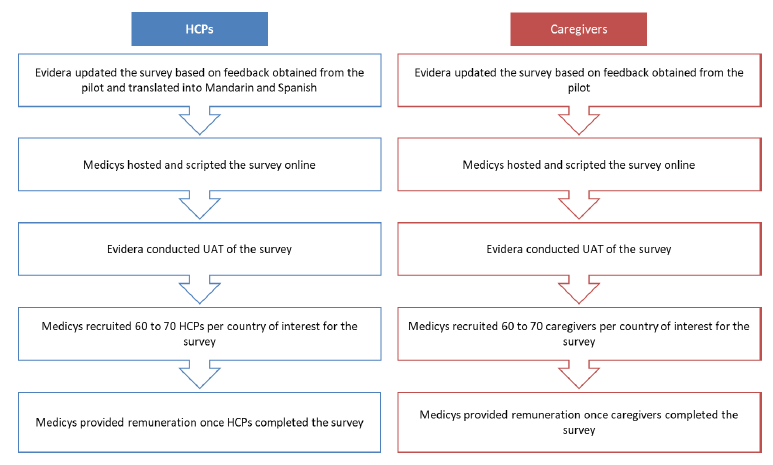


Abbreviations: HCP = healthcare professional; UAT = user acceptance testing

S5. Primary Endpoints Addressed in the Caregiver and HCP Surveys

| Objectives | Endpoints | Assessed in Caregiver or HCP Survey | Number of Questions in Survey |
| --- | --- | --- | --- |
| Identify and evaluate the burden of caring for an adult, adolescent, or child with epilepsy, from the caregiver’s perspective | Sociodemographic information | Caregiver | 8 |
|  | Questions about the PWE for whom caregivers provide care:   - Number of PWE for whom they care - How long they have been a caregiver for PWE - Gender of PWE for whom they care - Age of PWE for whom they care - Relation to PWE for whom they care - Type of seizures the PWE currently have - Number of seizure episodes the PWE have on average in a month - Time of day that most seizures occur - Assistance with eating, personal care, bowel and bladder management, mobility, transportation, communication, and activities at home, school, or work - Perceived level of assistance needed - Hours per week spent providing care to the PWE - Whether the PWE have been prescribed ASMs - If they take it - Reasons for not taking it - Usage of aids to take medication - Whether the PWE receive any therapies or treatments - Other medical conditions for which the PWE receive medication - Mental health and other difficulties of the PWE | Caregiver | 18 |
|  | Questions about how you would support the PWE:   - Sharing responsibilities to provide care for the PWE - Time dedicated to medical appointments for the PWE in the last 6 months - Main medical professional responsible for making decisions about the ASM - Questions about appointments - Information and knowledge about the condition - Support - Outlook of condition | Caregiver | 29 |
|  | Impacts related to caregiving duties:   - Physical impacts - Work/school impacts - Emotional impacts - Family impacts - Social impacts - Other impacts | Caregiver | 14 |
| Identify the perception and management of caregiver burden by HCPs, as well as the decision-making drivers in clinical practice for managing adults, adolescents, and children with epilepsy | Questions about clinical experience | HCP | 5 |
|  | Questions about PWE who are treated:   - Number of patients seen per week - Composition of PWE seen/treated - Proportion of patients with fully/partially controlled or refractory seizures - Proportion of adult, adolescent, and pediatric PWE who need significant support (i.e., ≥8 hours per day) from the caregiver - Activities patients need help with: eating, personal care, bowel and bladder management (e.g., using the toilet), transfer (e.g., from a bed to a chair), walking, communicating, activities at home, activities at school/work, taking medication, accompanying to medical appointments, events and/or hobbies, and unknown - Proportion of adult, adolescent, and pediatric PWE who need part-time support (i.e., <8 hours) - Activities patients need help with: eating, personal care, bowel and bladder management (e.g., using the toilet), transfer (e.g., from a bed to a chair), walking, communicating, activities at home, activities at school/work, taking medication, accompanying to medical appointments, events and/or hobbies, and unknown - Level of assistance required compared with other children without epilepsy - Questions about appointments - Questions about epilepsy medication - If they take it - Reasons for not taking it - Questions on seizure triggers and risk - Screening for depression and anxiety symptoms - Other difficulties of the PWE - Use of other therapies or treatments | HCP | 15 |
|  | Questions about caregivers and support needed:   - Caregiver roles - Sharing caregiving responsibilities - Appointments and reasons for caregiver nonattendance - Caregiver communication - Caregiver involvement in making treatment decisions - Topics of discussion - Difficulties and concerns faced by caregivers - Impact of caring for the PWE - Information and knowledge about epilepsy - Support | HCP | 14 |

Abbreviations: ASM = antiseizure medication; HCP = healthcare professional; PWE = persons with epilepsy

S6. Final Caregiver and HCP Surveys

**S7. Caregiver Level of Assistance Provided by Age Group**

| **Level of Assistance Caregiver Needed** | **Total (N=200)** | **Children**  **(0-11 years) (N=40)** | **Adolescents**  **(12-17 years)**  **(N=40)** | **Adults**  **(18-64 years)**  **(N=79)** | **Older Adults**  **(>65 years)**  **(N=41)** |
| --- | --- | --- | --- | --- | --- |
| **Eating, n (%)** | | | | | |
| Does not need help | 49 (24.5%) | 7 (17.5%) | 13 (32.5%) | 23 (29.1%) | 6 (14.6%) |
| Needs a little bit of help - supervision only | 86 (43.0%) | 16 (40.0%) | 20 (50.0%) | 37 (46.8%) | 13 (31.7%) |
| Needs some help | 34 (17.0%) | 9 (22.5%) | 2 (5.0%) | 12 (15.2%) | 11 (26.8%) |
| Needs a lot of help | 30 (15.0%) | 8 (20.0%) | 5 (12.5%) | 6 (7.6%) | 11 (26.8%) |
| Cannot do this at all without help | 1 (0.5%) | 0 (0.0%) | 0 (0.0%) | 1 (1.3%) | 0 (0.0%) |
| **Personal care, n (%)** | | | | | |
| Does not need help | 48 (24.0%) | 2 (5.0%) | 17 (42.5%) | 26 (32.9%) | 3 (7.3%) |
| Needs a little bit of help - supervision only | 71 (35.5%) | 17 (42.5%) | 18 (45.0%) | 24 (30.4%) | 12 (29.3%) |
| Needs some help | 47 (23.5%) | 9 (22.5%) | 1 (2.5%) | 23 (29.1%) | 14 (34.1%) |
| Needs a lot of help | 30 (15.0%) | 10 (25.0%) | 4 (10.0%) | 4 (5.1%) | 12 (29.3%) |
| Cannot do this at all without help | 4 (2.0%) | 2 (5.0%) | 0 (0.0%) | 2 (2.5%) | 0 (0.0%) |
| **Bowel and bladder management, n (%)** | | | | | |
| Does not need help | 72 (36.0%) | 11 (27.5%) | 21 (52.5%) | 33 (41.8%) | 7 (17.1%) |
| Needs a little bit of help - supervision only | 75 (37.5%) | 15 (37.5%) | 16 (40.0%) | 34 (43.0%) | 10 (24.4%) |
| Needs some help | 32 (16.0%) | 9 (22.5%) | 2 (5.0%) | 8 (10.1%) | 13 (31.7%) |
| Needs a lot of help | 18 (9.0%) | 3 (7.5%) | 1 (2.5%) | 3 (3.8%) | 11 (26.8%) |
| Cannot do this at all without help | 3 (1.5%) | 2 (5.0%) | 0 (0.0%) | 1 (1.3%) | 0 (0.0%) |
| **Mobility, n (%)** | | | | | |
| Does not need help | 92 (46.0%) | 22 (55.0%) | 25 (62.5%) | 34 (43.0%) | 11 (26.8%) |
| Needs a little bit of help - supervision only | 65 (32.5%) | 11 (27.5%) | 13 (32.5%) | 33 (41.8%) | 8 (19.5%) |
| Needs some help | 30 (15.0%) | 5 (12.5%) | 1 (2.5%) | 8 (10.1%) | 16 (39.0%) |
| Needs a lot of help | 11 (5.5%) | 1 (2.5%) | 1 (2.5%) | 3 (3.8%) | 6 (14.6%) |
| Cannot do this at all without help | 2 (1.0%) | 1 (2.5%) | 0 (0.0%) | 1 (1.3%) | 0 (0.0%) |
| **Communication, n (%)** | | | | | |
| Does not need help | 55 (27.5%) | 4 (10.0%) | 14 (35.0%) | 26 (32.9%) | 11 (26.8%) |
| Needs a little bit of help - supervision only | 78 (39.0%) | 16 (40.0%) | 19 (47.5%) | 36 (45.6%) | 7 (17.1%) |
| Needs some help | 46 (23.0%) | 15 (37.5%) | 6 (15.0%) | 14 (17.7%) | 11 (26.8%) |
| Needs a lot of help | 17 (8.5%) | 3 (7.5%) | 1 (2.5%) | 2 (2.5%) | 11 (26.8%) |
| Cannot do this at all without help | 4 (2.0%) | 2 (5.0%) | 0 (0.0%) | 1 (1.3%) | 1 (2.4%) |
| **Activities at home, school, or work, n (%)** | | | | | |
| Does not need help | 34 (17.0%) | 3 (7.5%) | 9 (22.5%) | 16 (20.3%) | 6 (14.6%) |
| Needs a little bit of help - supervision only | 87 (43.5%) | 15 (37.5%) | 24 (60.0%) | 36 (45.6%) | 12 (29.3%) |
| Needs some help | 56 (28.0%) | 16 (40.0%) | 5 (12.5%) | 23 (29.1%) | 12 (29.3%) |
| Needs a lot of help | 21 (10.5%) | 6 (15.0%) | 2 (5.0%) | 3 (3.8%) | 10 (24.4%) |
| Cannot do this at all without help | 2 (1.0%) | 0 (0.0%) | 0 (0.0%) | 1 (1.3%) | 1 (2.4%) |
| **Assistance needed by the child under care is more than other children, n (%)** | | | | | |
| Yes | 35 (17.5%) | 33 (82.5%) | 2 (5.0%) | 0 (0.0%) | 0 (0.0%) |
| No | 5 (2.5%) | 5 (12.5%) | 0 (0.0%) | 0 (0.0%) | 0 (0.0%) |
| I am not sure | 2 (1.0%) | 2 (5.0%) | 0 (0.0%) | 0 (0.0%) | 0 (0.0%) |
| Missing | 158 (79.0%) | 0 (0.0%) | 38 (95.0%) | 79 (100%) | 41 (100%) |
| **Hours per week spent caregiving, n (%)** | | | | | |
| ≤8 hours | 8 (4.0%) | 0 (0.0%) | 3 (7.5%) | 5 (6.3%) | 0 (0.0%) |
| 9-18 hours | 37 (18.5%) | 3 (7.5%) | 8 (20.0%) | 20 (25.3%) | 6 (14.6%) |
| 19-26 hours | 33 (16.5%) | 7 (17.5%) | 6 (15.0%) | 15 (19.0%) | 5 (12.2%) |
| 27-36 hours | 40 (20.0%) | 4 (10.0%) | 13 (32.5%) | 15 (19.0%) | 8 (19.5%) |
| 37-45 hours | 36 (18.0%) | 8 (20.0%) | 5 (12.5%) | 12 (15.2%) | 11 (26.8%) |
| ≥46 hours | 46 (23.0%) | 18 (45.0%) | 5 (12.5%) | 12 (15.2%) | 11 (26.8%) |

**S8. Burden of Caregiving and Medical Appointments by Age Group for which Care is Provided**

|  | **Total**  **(N=200)** | **Children**  **(0-11 years)**  **(n=40)** | **Adolescents**  **(12-17 years)**  **(n=40)** | **Adults**  **(18-64 years)**  **(n=79)** | **Older adults**  **(>65 years)**  **(n=41)** |
| --- | --- | --- | --- | --- | --- |
| **Caregiver assists person under care take their medicine, n (%)** | | | | | |
| Yes | 195 (97.5%) | 40 (100%) | 39 (97.5%) | 76 (96.2%) | 40 (97.6%) |
| No | 5 (2.5%) | 0 (0.0%) | 1 (2.5%) | 3 (3.8%) | 1 (2.4%) |
| **Hours per month spent on medical appointments, n (%)** | | | | |  |
| ≤2 hours | 15 (7.5%) | 1 (2.5%) | 3 (7.5%) | 6 (7.6%) | 5 (12.2%) |
| 3-4 hours | 55 (27.5%) | 7 (17.5%) | 12 (30.0%) | 27 (34.2%) | 9 (22.0%) |
| 5-6 hours | 42 (21.0%) | 6 (15.0%) | 7 (17.5%) | 22 (27.8%) | 7 (17.1%) |
| 7-8 hours | 40 (20.0%) | 15 (37.5%) | 7 (17.5%) | 10 (12.7%) | 8 (19.5%) |
| >8 hours | 48 (24.0%) | 11 (27.5%) | 11 (27.5%) | 14 (17.7%) | 12 (29.3%) |
| **Frequency of appointments with main medical professional, n (%)** | | | | |  |
| Monthly | 94 (47.0%) | 27 (67.5%) | 12 (30.0%) | 32 (40.5%) | 23 (56.1%) |
| Quarterly (every 3 months) | 86 (43.0%) | 13 (32.5%) | 25 (62.5%) | 37 (46.8%) | 11 (26.8%) |
| Twice a year | 8 (4.0%) | 0 (0.0%) | 0 (0.0%) | 5 (6.3%) | 3 (7.3%) |
| Yearly | 8 (4.0%) | 0 (0.0%) | 2 (5.0%) | 2 (2.5%) | 4 (9.8%) |
| Other (please specify)^a^ | 4 (2.0%) | 0 (0.0%) | 1 (2.5%) | 3 (3.8%) | 0 (0.0%) |
| **Take or travel with person under care to appointments, n (%)** | | | | |  |
| Yes, always | 177 (88.5%) | 38 (95.0%) | 36 (90.0%) | 66 (83.5%) | 37 (90.2%) |
| Yes, sometimes | 21 (10.5%) | 2 (5.0%) | 3 (7.5%) | 12 (15.2%) | 4 (9.8%) |
| No | 2 (1.0%) | 0 (0.0%) | 1 (2.5%) | 1 (1.3%) | 0 (0.0%) |
| **Sit in with person under care to appointments, n (%)** | | | | |  |
| Yes, always | 181 (90.5%) | 38 (95.0%) | 37 (92.5%) | 67 (84.8%) | 39 (95.1%) |
| Yes, sometimes | 16 (8.0%) | 2 (5.0%) | 2 (5.0%) | 10 (12.7%) | 2 (4.9%) |
| No | 3 (1.5%) | 0 (0.0%) | 1 (2.5%) | 2 (2.5%) | 0 (0.0%) |
| *If no, reasons for not attending appointments*^b^ |  |  |  |  |  |
| I do not have time to go to the appointment | 5 (26.3%) | 0 (0.0%) | 2 (66.7%) | 2 (16.7%) | 1 (50.0%) |
| The clinic does not allow me to attend the appointment | 2 (10.5%) | 0 (0.0%) | 0 (0.0%) | 2 (16.7%) | 0 (0.0%) |
| I want the person with epilepsy to be independent | 4 (21.1%) | 1 (50.0%) | 0 (0.0%) | 3 (25.0%) | 0 (0.0%) |
| The person with epilepsy requests that I do not go with them | 6 (31.6%) | 1 (50.0%) | 0 (0.0%) | 4 (33.3%) | 1 (50.0%) |
| Other reason^c^ | 6 (31.6%) | 1 (50.0%) | 1 (33.3%) | 4 (33.3%) | 0 (0.0%) |
| **Preparation for medical appointments, n (%)** | | | | |  |
| Arrange for transport | 101 (50.5%) | 23 (57.5%) | 19 (47.5%) | 37 (46.8%) | 22 (53.7%) |
| Prepare questions to ask the doctor | 166 (83.0%) | 34 (85.0%) | 30 (75.0%) | 70 (88.6%) | 32 (78.0%) |
| Prepare seizure diaries | 152 (76.0%) | 32 (80.0%) | 30 (75.0%) | 61 (77.2%) | 29 (70.7%) |
| Prepare medicine/prescriptions/ paperwork | 133 (66.5%) | 32 (80.0%) | 27 (67.5%) | 51 (64.6%) | 23 (56.1%) |
| Other preparations^d^ | 2 (1.0%) | 0 (0.0%) | 0 (0.0%) | 2 (2.5%) | 0 (0.0%) |
| I do not do anything to prepare for these appointments | 4 (2.0%) | 1 (2.5%) | 2 (5.0%) | 1 (1.3%) | 0 (0.0%) |

^a^ Other appointment frequencies include: 4 months; It depends on when you can get an appointment with the health insurance company, but 2 per year for sure if there hasn’t been a hospital; It depends, because in general it is 6 months, but if he has an epileptic seizure in the middle we go for a check-up and correction of the medication; The controls should be every 6 months but sometimes it is earlier depending on how he is; None.
^b^ Responses are not mutually exclusive.
^c^ Other reasons for not making appointment include: Because she is accompanied by my father who is her husband; She never goes alone; One of her last crises was in the street and from that moment on she never goes out alone; For work reasons I am unable to accompany; I accompany him most of the time; My child does not want to go to the doctor's office; The mother takes care; Work.
^d^ Other preparations include: I remind him that he must bring the results of the studies; His social security card and his ID card; None.

S9. Impacts Experienced by Caregivers by Country

| **Impact** | **Total (N=200)** | **Argentina (n=70)** | **Taiwan (n=65)** | **China (n=65)** |
| --- | --- | --- | --- | --- |
| **Emotional impacts experienced or worsened, n (%)^a^** | | | | |
| I have felt anxious | 117 (58.5%) | 39 (55.7%) | 34 (52.3%) | 44 (67.7%) |
| I have felt depressed | 91 (45.5%) | 38 (54.3%) | 30 (46.2%) | 23 (35.4%) |
| I have been in denial over the epilepsy diagnosis | 28 (14.0%) | 2 (2.9%) | 11 (16.9%) | 15 (23.1%) |
| I have felt frustrated | 80 (40.0%) | 29 (41.4%) | 26 (40.0%) | 25 (38.5%) |
| I have felt stressed | 133 (66.5%) | 43 (61.4%) | 57 (87.7%) | 33 (50.8%) |
| I have found it difficult coping | 39 (19.5%) | 22 (31.4%) | 14 (21.5%) | 3 (4.6%) |
| I have felt guilty | 52 (26.0%) | 11 (15.7%) | 30 (46.2%) | 11 (16.9%) |
| I have felt helpless | 65 (32.5%) | 27 (38.6%) | 23 (35.4%) | 15 (23.1%) |
| I have felt isolated | 26 (13.0%) | 10 (14.3%) | 11 (16.9%) | 5 (7.7%) |
| I have felt angry | 32 (16.0%) | 19 (27.1%) | 8 (12.3%) | 5 (7.7%) |
| I have felt fearful | 63 (31.5%) | 28 (40.0%) | 29 (44.6%) | 6 (9.2%) |
| I worry about future prospects for the PWE (education, marriage, children, friendships, family life, independence, etc.) | 89 (44.5%) | 34 (48.6%) | 40 (61.5%) | 15 (23.1%) |
| I have not experienced any emotional impacts due to caring for PWE | 5 (2.5%) | 4 (5.7%) | 0 (0.0%) | 1 (1.5%) |
| **Fatigue in the past 7 days, n (%)** | | | | |
| All the time | 43 (21.5%) | 18 (25.7%) | 14 (21.5%) | 11 (16.9%) |
| 3-5 days | 82 (41.0%) | 23 (32.9%) | 39 (60.0%) | 20 (30.8%) |
| <3 days | 62 (31.0%) | 18 (25.7%) | 12 (18.5%) | 32 (49.2%) |
| Not at all | 13 (6.5%) | 11 (15.7%) | 0 (0.0%) | 2 (3.1%) |
| **Sleep interruptions in the past 7 days, n (%)** | | | | |
| All the time | 26 (13.0%) | 13 (18.6%) | 6 (9.2%) | 7 (10.8%) |
| 3-5 days | 81 (40.5%) | 17 (24.3%) | 42 (64.6%) | 22 (33.8%) |
| <3 days | 72 (36.0%) | 24 (34.3%) | 15 (23.1%) | 33 (50.8%) |
| Not at all | 21 (10.5%) | 16 (22.9%) | 2 (3.1%) | 3 (4.6%) |
| **Impacts experienced as a result of caregiving, n (%)^a^** | | | | |
| I am not as productive at work or school | 80 (40.0%) | 19 (27.1%) | 28 (43.1%) | 33 (50.8%) |
| I lost my job | 16 (8.0%) | 6 (8.6%) | 4 (6.2%) | 6 (9.2%) |
| I have missed career/advancement opportunities | 28 (14.0%) | 17 (24.3%) | 8 (12.3%) | 3 (4.6%) |
| I changed my career path | 41 (20.5%) | 14 (20.0%) | 17 (26.2%) | 10 (15.4%) |
| I had to change my job | 39 (19.5%) | 14 (20.0%) | 14 (21.5%) | 11 (16.9%) |
| I had to adapt my working hours | 80 (40.0%) | 33 (47.1%) | 39 (60.0%) | 8 (12.3%) |
| I had to take a job that pays less | 19 (9.5%) | 10 (14.3%) | 3 (4.6%) | 6 (9.2%) |
| I am/have been under financial stress | 49 (24.5%) | 23 (32.9%) | 13 (20.0%) | 13 (20.0%) |
| I don’t have job security | 24 (12.0%) | 11 (15.7%) | 5 (7.7%) | 8 (12.3%) |
| Caring for PWE has not impacted my work/study | 24 (12.0%) | 14 (20.0%) | 4 (6.2%) | 6 (9.2%) |
| **Factors interfering with work/study, n (%)^a^** | | | | |
| Frequency of medical appointments | 82 (41.0%) | 34 (48.6%) | 19 (29.2%) | 29 (44.6%) |
| Duration of medical appointments | 65 (32.5%) | 12 (17.1%) | 26 (40.0%) | 27 (41.5%) |
| Patient’s dependency in completing activities of daily living | 137 (68.5%) | 35 (50.0%) | 56 (86.2%) | 46 (70.8%) |
| Frequency of medicine administration | 52 (26.0%) | 16 (22.9%) | 23 (35.4%) | 13 (20.0%) |
| Frequency of seizures | 81 (40.5%) | 17 (24.3%) | 40 (61.5%) | 24 (36.9%) |
| Severity of seizures | 79 (39.5%) | 13 (18.6%) | 39 (60.0%) | 27 (41.5%) |
| Risk of injury as a result of seizures | 66 (33.0%) | 16 (22.9%) | 25 (38.5%) | 25 (38.5%) |
| Cognitive and psychiatric effects of medicine | 39 (19.5%) | 15 (21.4%) | 13 (20.0%) | 11 (16.9%) |
| Other | 1 (0.5%) | 1 (1.4%) | 0 (0.0%) | 0 (0.0%) |
| **Difficulty enrolling patient under care in school, activity groups, or hobbies, n (%)** | | | | |
| Yes | 76 (38.0%) | 13 (18.6%) | 26 (40.0%) | 37 (56.9%) |
| There are no special instructors dedicated to children/adolescents/adults with special needs | 35 (46.1%) | 7 (53.8%) | 11 (42.3%) | 17 (45.9%) |
| There are a limited number of children/adolescents/adults with special needs that can be enrolled | 30 (39.5%) | 5 (38.5%) | 18 (69.2%) | 7 (18.9%) |
| Educational centers for children/adolescents/adults with special needs are too expensive | 35 (46.1%) | 5 (38.5%) | 16 (61.5%) | 14 (37.8%) |
| Educational centers for children/adolescents/adults with special needs are far from our home | 26 (34.2%) | 0 (0.0%) | 6 (23.1%) | 20 (54.1%) |
| No | 88 (44.0%) | 36 (51.4%) | 36 (55.4%) | 16 (24.6%) |
| Not applicable | 36 (18.0%) | 21 (30.0%) | 3 (4.6%) | 12 (18.5%) |
| **Coping strategies utilized, n (%)^a^** | | | | |
| I learned about the condition so I know what to do | 141 (70.5%) | 51 (72.9%) | 49 (75.4%) | 41 (63.1%) |
| I don't think about the future too much, I live in the moment | 47 (23.5%) | 6 (8.6%) | 23 (35.4%) | 18 (27.7%) |
| I do my best and leave the rest to the doctors | 130 (65.0%) | 27 (38.6%) | 61 (93.8%) | 42 (64.6%) |
| I have support from the medical team | 57 (28.5%) | 24 (34.3%) | 21 (32.3%) | 12 (18.5%) |
| I have my family to support me when I need it | 116 (58.0%) | 34 (48.6%) | 53 (81.5%) | 29 (44.6%) |
| I joined a support group | 16 (8.0%) | 9 (12.9%) | 4 (6.2%) | 3 (4.6%) |
| I take time out for myself to exercise/meditate/relax | 37 (18.5%) | 14 (20.0%) | 18 (27.7%) | 5 (7.7%) |
| My faith helps me cope | 35 (17.5%) | 9 (12.9%) | 24 (36.9%) | 2 (3.1%) |
| Other^b^ | 2 (1.0%) | 2 (2.9%) | 0 (0.0%) | 0 (0.0%) |

Abbreviation: PWE = persons with epilepsy

**^a^** Responses are not mutually exclusive.

^b^ Other includes therapy via Zoom and a first aid course to learn how to act during seizures.

S10. Caregiver Work and Financial Impacts

S11. Factors Interfering with Work/Study as Reported by Caregivers

S12. Fatigue Experience as Reported by Caregivers

S13. Sleep Experience as Reported by Caregivers

S14. Difficulty Enrolling PWE in Activities as Reported by Caregivers


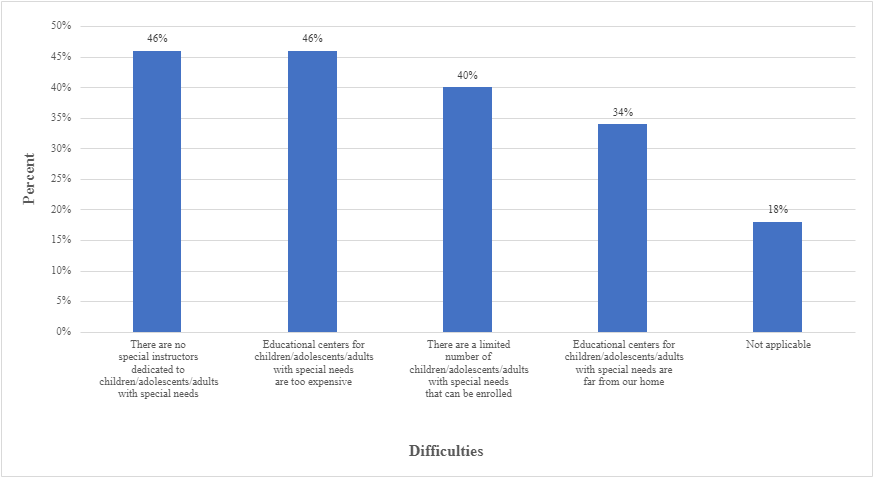


S15. Coping Strategies as Reported by Caregivers

S16. Information Relevance and Sources of Support for Caregivers by Country

| **Type** | **Total (N=200)** | **Argentina (n=70)** | **Taiwan (n=65)** | **China (n=65)** |
| --- | --- | --- | --- | --- |
| **Types of information most relevant to caregiver, n (%)^a^** | | | | |
| Information about epilepsy (e.g., what the patient experiences during seizure, what to do during a seizure) | 184 (92.0%) | 59 (84.3%) | 63 (96.9%) | 62 (95.4%) |
| Information about treatments (e.g., treatment options, side effects, pros and cons) | 156 (78.0%) | 45 (64.3%) | 63 (96.9%) | 48 (73.8%) |
| Information about support services for education for the PWE | 85 (42.5%) | 28 (40.0%) | 30 (46.2%) | 27 (41.5%) |
| Information about psychological support services for the PWE | 109 (54.5%) | 37 (52.9%) | 38 (58.5%) | 34 (52.3%) |
| Information about psychological support services for me | 99 (49.5%) | 41 (58.6%) | 37 (56.9%) | 21 (32.3%) |
| Information about available support groups | 70 (35.0%) | 25 (35.7%) | 28 (43.1%) | 17 (26.2%) |
| Information about services to care for PWE | 95 (47.5%) | 22 (31.4%) | 48 (73.8%) | 25 (38.5%) |
| Information about legal questions related to epilepsy (e.g., driving, work) | 45 (22.5%) | 18 (25.7%) | 22 (33.8%) | 5 (7.7%) |
| Information about support services for work options for the PWE | 43 (21.5%) | 18 (25.7%) | 19 (29.2%) | 6 (9.2%) |
| Information about available financial support | 62 (31.0%) | 23 (32.9%) | 30 (46.2%) | 9 (13.8%) |
| **Satisfaction with the information provided, n (%)** | | | | |
| Extremely dissatisfied | 3 (1.5%) | 2 (2.9%) | 0 (0.0%) | 1 (1.5%) |
| Quite dissatisfied | 8 (4.0%) | 4 (5.7%) | 2 (3.1%) | 2 (3.1%) |
| Neither dissatisfied nor satisfied | 76 (38.0%) | 19 (27.1%) | 21 (32.3%) | 36 (55.4%) |
| Quite satisfied | 110 (55.0%) | 42 (60.0%) | 42 (64.6%) | 26 (40.0%) |
| Extremely satisfied | 3 (1.5%) | 3 (4.3%) | 0 (0.0%) | 0 (0.0%) |
| **Sources of emotional support, n (%)^a^** | | | | |
| Medical professionals | 73 (36.5%) | 26 (37.1%) | 27 (41.5%) | 20 (30.8%) |
| Family members or friends | 138 (69.0%) | 48 (68.6%) | 51 (78.5%) | 39 (60.0%) |
| Epilepsy support groups | 30 (15.0%) | 12 (17.1%) | 11 (16.9%) | 7 (10.8%) |
| Mental health support groups | 35 (17.5%) | 6 (8.6%) | 15 (23.1%) | 14 (21.5%) |
| Religious groups | 32 (16.0%) | 5 (7.1%) | 26 (40.0%) | 1 (1.5%) |
| Other**^b^** | 1 (0.5%) | 1 (1.4%) | 0 (0.0%) | 0 (0.0%) |
| **Sources of financial support, n (%)^a^** | | | | |
| Medical professionals | 7 (3.5%) | 3 (4.3%) | 0 (0.0%) | 4 (6.2%) |
| Family members or friends | 76 (38.0%) | 18 (25.7%) | 20 (30.8%) | 38 (58.5%) |
| Epilepsy support groups | 11 (5.5%) | 2 (2.9%) | 0 (0.0%) | 9 (13.8%) |
| Mental health support groups | 6 (3.0%) | 1 (1.4%) | 0 (0.0%) | 5 (7.7%) |
| Religious groups | 5 (2.5%) | 2 (2.9%) | 2 (3.1%) | 1 (1.5%) |
| I don't reach out to anyone for emotional or moral support | 2 (1.0%) | 0 (0.0%) | 1 (1.5%) | 1 (1.5%) |
| Other**^c^** | 1 (0.5%) | 1 (1.4%) | 0 (0.0%) | 0 (0.0%) |
| **Sources of support with decisions and treatment, n (%)^a^** | | | | |
| Medical professionals | 121 (60.5%) | 30 (42.9%) | 51 (78.5%) | 40 (61.5%) |
| Family members or friends | 73 (36.5%) | 18 (25.7%) | 36 (55.4%) | 19 (29.2%) |
| Epilepsy support groups | 28 (14.0%) | 5 (7.1%) | 10 (15.4%) | 13 (20.0%) |
| Mental health support groups | 10 (5.0%) | 3 (4.3%) | 1 (1.5%) | 6 (9.2%) |
| Religious groups | 3 (1.5%) | 1 (1.4%) | 2 (3.1%) | 0 (0.0%) |
| **Sources of practical support, n (%)^a^** | | | | |
| Medical professionals | 52 (26.0%) | 9 (12.9%) | 25 (38.5%) | 18 (27.7%) |
| Family members or friends | 65 (32.5%) | 17 (24.3%) | 22 (33.8%) | 26 (40.0%) |
| Epilepsy support groups | 11 (5.5%) | 3 (4.3%) | 1 (1.5%) | 7 (10.8%) |
| Mental health support groups | 11 (5.5%) | 2 (2.9%) | 3 (4.6%) | 6 (9.2%) |
| Religious groups | 2 (1.0%) | 0 (0.0%) | 1 (1.5%) | 1 (1.5%) |
| **Sources of other support, n (%)^a^** | | | | |
| Medical professionals | 1 (0.5%) | 0 (0.0%) | 1 (1.5%) | 0 (0.0%) |
| Family members or friends | 2 (1.0%) | 2 (2.9%) | 0 (0.0%) | 0 (0.0%) |
| I don't reach out to anyone for emotional or moral support | 2 (1.0%) | 2 (2.9%) | 0 (0.0%) | 0 (0.0%) |

**^a^** Responses are not mutually exclusive.

**^b^** Other includes I don’t know, I have no help, none, sometimes siblings and children.

^c^ Other includes medical insurance.

S17. Patient Assistance and Support Needs by Age Group and Country

| **Characteristic** | **Total (n=60)** | **Argentina (n=18)** | **Taiwan (n=14)** | **China (n=28)** |
| --- | --- | --- | --- | --- |
| **Proportion of pediatric patients needing significant support (%)** | | | | |
| n | 60 | 18 | 14 | 28 |
| Mean (SD) | 49.6 (32.2) | 49.8 (35.2) | 28.4 (29.2) | 60.2 (26.9) |
| Median | 50.0 | 55.0 | 17.5 | 55.0 |
| Range (min-max) | (0-100) | (1-100) | (0-90) | (5-100) |
| **Type of significant support needed by pediatric patients** | | | | |
| Eating | 41 (68.3%) | 15 (83.3%) | 8 (57.1%) | 18 (64.3%) |
| Personal care | 48 (80.0%) | 17 (94.4%) | 10 (71.4%) | 21 (75.0%) |
| Bowel and bladder management (e.g., using the toilet) | 35 (58.3%) | 13 (72.2%) | 11 (78.6%) | 11 (39.3%) |
| Transfer (e.g., from a bed to a chair) | 28 (46.7%) | 13 (72.2%) | 7 (50.0%) | 8 (28.6%) |
| Walking | 27 (45.0%) | 13 (72.2%) | 6 (42.9%) | 8 (28.6%) |
| Communicating | 36 (60.0%) | 11 (61.1%) | 8 (57.1%) | 17 (60.7%) |
| Activities at home | 34 (56.7%) | 10 (55.6%) | 9 (64.3%) | 15 (53.6%) |
| Activities at school/work | 28 (46.7%) | 12 (66.7%) | 7 (50.0%) | 9 (32.1%) |
| Taking medication | 48 (80.0%) | 18 (100.0%) | 11 (78.6%) | 19 (67.9%) |
| Accompanying to medical appointments, events and/or hobbies | 41 (68.3%) | 16 (88.9%) | 8 (57.1%) | 17 (60.7%) |
| Unknown | 2 (3.3%) | 0 (0.0%) | 1 (7.1%) | 1 (3.6%) |
| **Proportion of adolescent patients needing significant support (%)** | | | | |
| n | 131 | 44 | 36 | 51 |
| Mean (SD) | 24.7 (22.5) | 24.5 (23.6) | 13.3 (14.1) | 32.9 (23.0) |
| Median | 20.0 | 15.0 | 10.0 | 21.0 |
| Range (min-max) | (0-100) | (1-100) | (0-50) | (5-80) |
| **Type of significant support needed by adolescent patients** | | | | |
| Eating | 59 (45.0%) | 21 (47.7%) | 18 (50.0%) | 20 (39.2%) |
| Personal care | 75 (57.3%) | 28 (63.6%) | 17 (47.2%) | 30 (58.8%) |
| Bowel and bladder management (e.g., using the toilet) | 44 (33.6%) | 15 (34.1%) | 17 (47.2%) | 12 (23.5%) |
| Transfer (e.g., from a bed to a chair) | 50 (38.2%) | 20 (45.5%) | 19 (52.8%) | 11 (21.6%) |
| Walking | 49 (37.4%) | 14 (31.8%) | 19 (52.8%) | 16 (31.4%) |
| Communicating | 67 (51.1%) | 21 (47.7%) | 22 (61.1%) | 24 (47.1%) |
| Activities at home | 58 (44.3%) | 19 (43.2%) | 16 (44.4%) | 23 (45.1%) |
| Activities at school/work | 64 (48.9%) | 18 (40.9%) | 20 (55.6%) | 26 (51.0%) |
| Taking medication | 98 (74.8%) | 42 (95.5%) | 26 (72.2%) | 30 (58.8%) |
| Accompanying to medical appointments, events and/or hobbies | 92 (70.2%) | 37 (84.1%) | 17 (47.2%) | 38 (74.5%) |
| Unknown | 3 (2.3%) | 0 (0.0%) | 3 (8.3%) | 0 (0.0%) |
| **Proportion of adult patients needing significant support (%)** | | | | |
| n | 182 | 55 | 63 | 64 |
| Mean (SD) | 28.3 (20.7) | 29.5 (23.0) | 23.7 (17.7) | 31.7 (20.8) |
| Median | 20.0 | 20.0 | 20.0 | 30.0 |
| Range (min-max) | (0-100) | (1-100) | (0-80) | (5-85) |
| **Type of significant support needed by adult patients** | | | | |
| Eating | 103 (56.6%) | 26 (47.3%) | 45 (71.4%) | 32 (50.0%) |
| Personal care | 130 (71.4%) | 35 (63.6%) | 50 (79.4%) | 45 (70.3%) |
| Bowel and bladder management (e.g., using the toilet) | 94 (51.6%) | 15 (27.3%) | 49 (77.8%) | 30 (46.9%) |
| Transfer (e.g., from a bed to a chair) | 99 (54.4%) | 21 (38.2%) | 49 (77.8%) | 29 (45.3%) |
| Walking | 86 (47.3%) | 17 (30.9%) | 41 (65.1%) | 28 (43.8%) |
| Communicating | 80 (44.0%) | 24 (43.6%) | 31 (49.2%) | 25 (39.1%) |
| Activities at home | 85 (46.7%) | 28 (50.9%) | 29 (46.0%) | 28 (43.8%) |
| Activities at school/work | 44 (24.2%) | 17 (30.9%) | 13 (20.6%) | 14 (21.9%) |
| Taking medication | 141 (77.5%) | 49 (89.1%) | 48 (76.2%) | 44 (68.8%) |
| Accompanying to medical appointments, events, and/or hobbies | 129 (70.9%) | 46 (83.6%) | 35 (55.6%) | 48 (75.0%) |
| Unknown | 3 (1.6%) | 1 (1.8%) | 2 (3.2%) | 0 (0.0%) |

Abbreviations: max = maximum; min = minimum; SD = standard deviation

S18. HCP Recommended Sources of Information for Caregivers and Patients Overall (N=200)

| **Source** | **Source at Diagnosis** | **When Starting a Treatment** | **When Exploring a New Treatment** | **When New Concerns Arose** |
| --- | --- | --- | --- | --- |
| **Sources of information to learn about epilepsy** | | | | |
| Facebook groups or other closed online groups (n=50) | 26 (52.0%) | 21 (42.0%) | 14 (28.0%) | 15 (30.0%) |
| Written information (e.g., printed leaflets) (n=128) | 90 (70.3%) | 82 (64.1%) | 50 (39.1%) | 41 (32.0%) |
| Oral information (e.g., during medical appointments/visits) (n=168) | 126 (75.0%) | 117 (69.6%) | 84 (50.0%) | 72 (42.9%) |
| Support groups (online or face to face) (n=54) | 25 (46.3%) | 25 (46.3%) | 13 (24.1%) | 24 (44.4%) |
| Other people who care for PWE (n=54) | 18 (33.3%) | 24 (44.4%) | 19 (35.2%) | 21 (38.9%) |
| Other people with epilepsy (n=42) | 11 (26.2%) | 18 (42.9%) | 13 (31.0%) | 21 (50.0%) |
| A named person from the epilepsy team from the hospital, like a nurse (n=55) | 27 (49.1%) | 38 (69.1%) | 25 (45.5%) | 20 (36.4%) |
| YouTube/social media videos (n=44) | 19 (43.2%) | 20 (45.5%) | 24 (54.5%) | 23 (52.3%) |
| An informal place where they can meet others in the same situation as them (n=19) | 10 (52.6%) | 8 (42.1%) | 7 (36.8%) | 9 (47.4%) |
| Other**^b^** (n=4) | 3 (75.0%) | 1 (25.0%) | 1 (25.0%) | 1 (25.0%) |
| **Sources of information to support caregiving** | | | | |
| General internet searches (n=49) | 38 (77.6%) | 18 (36.7%) | 15 (30.6%) | 14 (28.6%) |
| Facebook groups or other closed online groups (n=41) | 23 (56.1%) | 15 (36.6%) | 9 (22.0%) | 17 (41.5%) |
| Written information (e.g., printed leaflets) (n=124) | 99 (79.8%) | 80 (64.5%) | 40 (32.3%) | 32 (25.8%) |
| Oral information (e.g., during medical appointments/visits) (n=149) | 119 (79.9%) | 90 (60.4%) | 63 (42.3%) | 63 (42.3%) |
| Support groups (online or face to face) (n=90) | 49 (54.4%) | 38 (42.2%) | 41 (45.6%) | 42 (46.7%) |
| Other people who care for PWE (n=69) | 35 (50.7%) | 25 (36.2%) | 21 (30.4%) | 34 (49.3%) |
| Other PWE (n=42) | 19 (45.2%) | 18 (42.9%) | 17 (40.5%) | 23 (54.8%) |
| A named person from the epilepsy team from the hospital, like a nurse (n=84) | 43 (51.2%) | 50 (59.5%) | 30 (35.7%) | 39 (46.4%) |
| YouTube/social media videos (n=37) | 21 (56.8%) | 17 (45.9%) | 22 (59.5%) | 20 (54.1%) |
| An informal place where they can meet others in the same situation as them (n=29) | 12 (41.4%) | 11 (37.9%) | 10 (34.5%) | 20 (69.0%) |
| Other**^c^** (n=1) | 1 (100.0%) | 1 (100.0%) | 1 (100.0%) | 1 (100.0%) |

Abbreviation: PWE = persons with epilepsy

**^a^** Responses are not mutually exclusive.

**^b^** Other includes an app called Ainsoph that is about to be released for PWE from different parts of the world to share their experiences, health education articles online, ILAE for patients, and patients’ association.

**^c^** Other includes ongoing academic training.
